# Optimizing genomic selection models for wheat breeding under contrasting water regimes in a mediterranean environment

**DOI:** 10.1186/s13007-025-01467-5

**Published:** 2025-12-05

**Authors:** Venkata Rami Reddy Yannam, Marta S. Lopes, Jose Miguel Soriano

**Affiliations:** 1 Sustainable Field Crops Program, Institute for Food and Agricultural Research and Technology (IRTA), Lleida, Spain; 2 Department of Agricultural and Forest Sciences and Engineering, University of Lleida, Av. Alcalde Rovira Roure 191, Lleida, 25198 Spain; 3 AGROTECNIO-CERCA Center, Av. Alcalde Rovira Roure 191, Lleida, 25198 Spain

**Keywords:** Genomic selection, Wheat breeding, Water stress, Machine learning models

## Abstract

**Background:**

Bread wheat (*Triticum aestivum* L.) is a vital global crop, supplying 20% of the protein in the human diet. Improving its productivity and resilience, particularly under water-limited conditions, is a major breeding priority. Genomic selection offers a promising approach to accelerate genetic gains by predicting complex traits using genome-wide marker data. This study evaluated the performance of various genomic selection (GS) models in predicting key agronomic traits under contrasting well-watered (WW) and water-stressed (WS) conditions, with the goal of enhancing drought adaptation in wheat breeding programs.

**Results:**

A panel of 179 wheat lines was evaluated for grain yield, yield components, and grain protein content. Models were trained on data from well-watered and water-stressed regimes independently, as well as on combined data from both conditions. Predictive approaches included linear models (Ridge Regression Best Linear Unbiased Prediction and Bayesian methods), semi-parametric models (Reproducing Kernel Hilbert Space Regression), and machine learning algorithms (Random Forest, Support Vector Machine, and Extreme Gradient Boosting). Ridge regression consistently delivered strong performance across all traits and conditions, with mean *r*_*MG*_ of 0.70 (water-stressed), 0.64 (well-watered), and 0.65 (combined). Machine learning models, especially Random Forest and Extreme Gradient Boosting, performed competitively for complex traits such as grain yield and thousand kernel weight. Random Forest achieved a *r*_*MG*_ of 0.81 for grain yield and 0.73 for thousand kernel weight under well-watered conditions. Trait stability was observed under well-watered conditions for thousand kernel weight and plant height, supported by moderate heritability estimates (0.69–0.74). Cross-validation comparisons showed consistent model performance across validation schemes, with leave-one-out cross-validation offering slightly improved accuracy for select traits and models. Notably, models trained under water-stressed conditions generalized better when tested on well-watered data than the reverse, highlighting the value of diverse training environments.

**Conclusions:**

Genomic selection models, particularly ridge regression and machine learning approaches, demonstrated reliable predictive performance across environments and traits. Incorporating data from multiple environmental conditions improves prediction accuracy and supports the development of drought-resilient wheat lines. These results reinforce the utility of genomic selection in modern wheat breeding programs for enhancing both productivity and stress tolerance.

**Supplementary Information:**

The online version contains supplementary material available at 10.1186/s13007-025-01467-5.

## Background

Bread wheat (*Triticum aestivum* L.), provides a substantial portion of human calories and proteins, highlighting its nutritional and economic significance. Enhancing wheat production is crucial for global food security amidst population growth and climate challenges. Despite its adaptability, wheat is significantly influenced by environmental factors such as temperature and water availability, and biotic stresses. Increasing drought frequency and severity due to climate change undermines yields, necessitating the development of new drought-tolerant varieties. Research has emphasized breeding strategies for water stress resilience, as traditional varieties often fail under changing climatic conditions [1, 2].

New efficient strategies combining molecular approaches with accelerated development of elite germplasm are needed to accelerate the development and delivery of improved germplasm. However, traditional QTL mapping has limitations such as the inconsistency of identified QTLs across environments, as environmental factors can influence gene expression, making QTLs unreliable for breeding applications [3, 4], while marker-assisted selection (MAS) extensively uses major genes for genotype selection. Thus, MAS becomes an ineffective strategy for improving complex traits controlled by multiple loci with minor effects, epistasis and genotype by environment interactions (GEI). In this aspect, genomic selection (GS) allows the prediction of breeding values using whole-genome marker data, capturing large-effect QTLs and small-effect loci, explaining more genetic variance, and enhancing genomic estimated breeding value (GEBV) accuracy. GS modifies MAS by calculating whole-genome marker effects for GEBVs, increasing prediction accuracy by 28% in multifamily wheat populations compared with MAS [5–7].

GS allows breeders to predict genotype performance using genetic data, enhancing breeding efficiency, particularly in varied environments where traditional phenotypic selection is laborious and expensive. Advanced predictive models, such as machine learning (ML) techniques, adeptly capture complex GEI affecting trait expression and facilitate the selection of high-performing drought-resistant genotypes [8, 9]. The integration of genomic data with sophisticated statistical models has been shown to improve prediction accuracy, making it a crucial component of contemporary breeding programs [10].

Cross-validation evaluates genomic selection (GS) effectiveness by splitting genotyped and phenotyped lines into training (TS) and validation sets (VS). The TS estimates marker effects to predict GEBVs for the VS, with predictive ability (*r*_*MP*_) measured by the correlation between GEBVs and observed phenotypes [11]. Prediction accuracy (*r*_*MG*_) is calculated as *r*_*MP*_/√h², reflecting success relative to phenotypic selection [12, 13]. Two methods are commonly used: leave-one-out cross-validation (LOOCV), where each genotype is tested once while maximizing training data, and k-fold cross-validation (KFCV), which splits data into k parts for iterative training and testing [14, 15]. LOOCV is effective for small datasets but computationally intensive and prone to overfitting [16]. KFCV, with an 80:20 training-to-testing split, balances training size and generalization, providing robust predictive accuracy [6–18]. Both methods aid in evaluating GS models for traits like drought tolerance and grain yield in wheat breeding, enhancing efforts to develop climate-resilient crops [19].

Extensive research has been conducted on GS across various major cereal species, with a notable emphasis on maize [20–25], wheat [26–29] and rice [30–32]. Multiple models, including GBLUP, Bayes A, Bayes B, Bayes C, and Bayes Cpi, have been implemented with inconsistent results. While a specific model may demonstrate superior performance for one trait, it may not exhibit comparable efficacy for another [5, 7]. Sandhu et, al. [33] reported that ML models, achieved 1–5% higher prediction accuracy compared to traditional BLUP-based and Bayesian models for quality traits in bread wheat. Similarly, comparable results were noted by [34–36] in predicting quantitative traits in wheat (including yield, plant growth, plant height, grain characteristics, and protein content), emphasising the need for further exploration of machine and deep learning models due to their enhanced accuracy. Additionally, k-fold cross-validation has been shown to provide a more robust estimate of model performance than LOOCV, as it reduces the variance associated with the evaluation of model accuracy by averaging results across multiple subsets of data [14, 37].

GS accuracy in varying water conditions hinges on genotypic diversity and drought resistance. Genotypes performing well under both optimal and drought conditions show higher stress tolerance indices, crucial for selecting high-yield cultivars across environments [38]. GS accuracy depends on trait architecture, heritability, TS size and composition, marker density, and the statistical model for estimating marker effects [39]. These elements are crucial for developing drought-tolerant wheat varieties to tackle climate change. The TS, with known phenotypic and genotypic data, is essential for training predictive models, thereby speeding up the selection process for new breeding lines. The TS’ quality and diversity are critical for capturing extensive genetic variations [27, 40]. Genotyping accuracy, statistical methods, TS size, and environmental conditions during phenotypic data collection significantly affect GS effectiveness [6, 17, 41, 42]. Addressing these factors enhances GS robustness and reliability, fostering more efficient breedingprograms.

This study evaluated the performance of genomic selection models, including RR-BLUP, Bayesian approaches, and ML models (SVM, RF, and XGB), within a TS using k-fold and LOOCV. Cross-condition training and testing analyses were conducted to assess model performance when trained under WW conditions and tested under WS conditions, and vice versa, to identify trends in model robustness and predictive accuracy across different environmental conditions, focusing on models trained and tested under the same conditions.

## Methods

### Plant material and experimental design

A total of 179 modern bread wheat (*Triticum aestivum* L.) lines comprising commercial cultivars used as parental lines in the Institute for Food and Agricultural Research and Technology (IRTA) breeding program, and breeding selections at different generations (F6 to F10) were used for developing prediction models. The germplasm was evaluated over two consecutive growing seasons (20/21 and 21/22) under two contrasting water regimes: rainfed (water stress, WS) and irrigated (well-watered, WW) conditions (Table 1). In the well-watered (WW) treatment, irrigation was scheduled to avoid any visible water stress and to maintain soil moisture near field capacity throughout the growing season, supplementing natural rainfall with sprinkler irrigation as needed. Total irrigation volumes for WW plots in each season are reported in (Table 1). Field trials were conducted in Almacelles (coordinates: 41°43′54″ N, 0°25′24″ E, elevation: 221 m) during the first season and Sucs (coordinates: 41°41′41″ N, 0°25′35″ E, elevation: 284 m) during the second season, using a randomised complete block design (RCBD) with two replications per water regime (i.e., two complete blocks, block size = 200 plots, one plot per genotype, no designated checks, with border rows sown with ‘Artur Nick’). These locations, positioned near Lleida. Spain and approximately 4 km apart, experience a characteristic Mediterranean climate. Throughout the trials, optimal fertilization was maintained, and locally approved pesticides were used to manage pests and diseases effectively. Each plot measured 2.5 m × 1.2 m (3.0 m²) within a field of 86.5 m × 27 m. Field data were collected in two environments, Almacelles (2020/21) and Sucs (2021/22), with both water regimes (WW, WS) evaluated in each. Although 200 entries were planted per block, 21 were excluded after quality check QC for genotyping/phenotypic issues, leaving 179 lines for prediction analyses. During both growing seasons, nitrogen (N) fertilizer was not applied, as soil tests revealed an excess of 250 kg ha⁻¹ of N due to historical overfertilization. However, in the 21/22 cycle, a pre-sowing application of 27 kg ha⁻¹ of N was made using diammonium phosphate (18–46).

**Table 1:** Comparison of meteorological parameters between 2021 and 2022

Month	Avg SR	Total PP	Avg T Min	Avg T Max	Avg T Mean	Avg ET₀	Total IRR
Dec-20	6.06	30.70	2.05	10.10	5.73	0.66	0
Jan-21	7.47	58.20	-0.68	9.24	3.89	0.81	0
Feb-21	9.35	14.60	4.95	15.22	9.69	1.25	10
Mar-21	17.25	5.90	2.90	17.96	10.10	2.55	26
Apr-21	18.49	29.70	5.82	19.07	12.06	2.90	81
May-21	25.90	20.50	9.83	24.43	17.07	4.54	85
Jun-21	25.26	19.60	14.97	30.19	22.18	4.92	30
Jul-21	27.55	64.40	16.64	33.14	24.41	5.49	0
**Average/Sum**	**17.16**	**243.60**	**7.06**	**19.92**	**13.14**	**2.89**	**232**
Dec-21	3.44	3.40	2.96	8.64	5.56	0.44	0
Jan-22	9.35	4.00	-2.77	10.06	2.78	0.90	30
Feb-22	11.70	3.30	1.68	15.62	8.11	1.51	20
Mar-22	10.77	39.20	5.21	15.53	10.02	1.64	0
Apr-22	19.67	52.80	6.23	20.01	12.84	3.15	50
May-22	25.49	9.00	11.76	29.11	19.92	4.83	140
Jun-22	27.26	13.90	16.80	33.90	24.90	5.56	60
Jul-22	27.31	27.30	18.26	35.93	26.50	5.57	0
**Average/Sum**	**16.87**	**152.90**	**7.51**	**21.10**	**13.83**	**2.95**	**300.00**

Avg SR (MJ m⁻²): Average of Solar radiation/month; Total PP (mm): Sum of Precipitation/month; Avg T Mean (°C): Average temperature/month; Avg T Max (°C): Average of Maximum temperature/month; Avg T Min (°C): Average of Minimum temperature/month; Avg ET₀ (mm): Average Reference evapotranspiration; Total IRR (mm): Total Irrigation applied/month; The AVERAGE/SUM row represents the average for temperature values, solar radiation, evapotranspiration, and the total sum for precipitation, irrigation over the recorded months

For well water trails (WW), sowing took place on 04/12/20 (Crop cycle 20–21) and 15/12/21 (Crop cycle 21–22), with harvest on 29/07/21 and 27/07/22, respectively

For water-stress trails (WS), sowing occurred on 03/12/20 (Crop cycle 20–21) and 15/12/21 (Crop cycle 21–22), with harvest on 23/07/21 and 22/07/22. Dates are in DD/MM/YY format

### Phenotypic data

Phenotypic data were collected over two years to assess agronomic performance. Experimental plots comprised eight rows (3 m^2^ × 0.15 m spacing) with a sowing density of 250 germinable seeds m². At physiological maturity (GS87), plant height (PH, cm) was measured on three main stems per plot. Before harvest, a 1-meter section in the central row was sampled to determine spikes per square meter (NSm²), while grains per spike (NGS) were assessed from 20 spikes per genotype. Grain yield (GY, t ha⁻¹) was measured from the entire 3 m^2^ plot at 12% moisture after mechanical harvesting. Thousand-kernel weight (TKW, g) was calculated from a random 10 g grain sample, and grain protein content (GP, %) was analyzed using an NIR Systems 6500 (Foss, Denmark). Trait means from two replicates were used for statistical analyses.

### Genotypic data

The population was genotyped with 24,145 SNP markers using the Illumina Infinium 25 K Wheat SNP Array at SGS INSTITUT FRESENIUS GmbH (Trait Genetics Section, Gatersleben, Germany). A quality filtering pipeline was implemented using R software to ensure the accuracy and reliability of the marker data. Markers with a minor allele frequency (MAF) lower than 5% were excluded from the dataset. Additionally, markers with more than 30% missing data were discarded, and all heterozygous markers were removed. Missing data in markers with less than 30% missing values were imputed using the VIM: Visualization and Imputation of Missing Values R package, following the k-Nearest Neighbour Imputation (KNN) (K = 5) method described by Kowarik et al. [43]. This choice of imputation aligns with common wheat GS practice that relies on LD-kNNi in TASSEL/LinkImpute and performs robustly for prediction when rare variants are filtered (e.g., MAF ≥ 0.05 in our case) [44–46]. After the complete filtering process, 20,223 SNPs remained and were used for genomic prediction analyses.

### Statistical analysis

Trait correlation analyses were performed using the “*sjPlot*” package in R [47]. Genotypic variance (GV), phenotypic variance (PV), environmental variance (EV), genotypic coefficients of variation (GCV), phenotypic coefficient of variation (PCV), environmental coefficient of variance (ECV), estimated using the PROC MIXED procedure in SAS OnDemand for Academics separately for WW and WS, plot-level data were analyzed by linear mixed models fitted by REML [48], For each water regime (WW, WS), we fitted a linear mixed model across the two years by REML in SAS PROC MIXED is as follows:

$$\:{\mathcal{Y}}_{ikj}=\mu\:+{g}_{i}+{\mathcal{Y}}_{k}+\left(gy\right)ik+bj\left(k\right)+{\epsilon\:}_{ikj}$$

where genotype $\:{g}_{i}$ *~ N(0*, $\:{\sigma\:}_{g}^{2}$*)*, year $\:{y}_{k}$ *~ N(0*, $\:{\sigma\:}_{y}^{2}$*)*, genotype×year *(gy)ik ~ N(0*,* σgy*^*2*^*)*, block nested within year $\:{b}_{j\left(k\right)}$ *~ N(0*, $\:{\sigma\:}_{b}^{2}$*)*, and $\:{\epsilon\:}_{ikj}$ *~ N(0*, $\:{\sigma\:}_{e}^{2}$*)*. Broad-sense heritability (^2^) was estimated for each treatment using the formula:

$$\:{H}^{2}=\frac{{\sigma\:}_{g}^{2}}{{\sigma\:}_{g}^{2}+\frac{{\sigma\:}_{g\times\:e}^{2}}{e}+\frac{{\sigma\:}_{e}^{2}}{re}}$$

where $\:{\sigma\:}_{g}^{2}$ represents the genetic variance and $\:{\sigma\:}_{e}^{2}$ denotes the residual error variance, $\:{\sigma\:}_{g\times\:e}^{2}$ is the GEI variance (interaction of genotype and year variance); $\:e$ is the number of years; *r* is the number of replicates per year.

## Genomic selection models

The performance of ten prediction models (seven parametric and three non-parametric models) was assessed for all six traits evaluated in this study. The parametric models included RRBLUP, Bayes B, Bayes A, Bayes Lasso, Bayes Ridge Regression (BRR), Bayes C, and Reproducing Kernel Hilbert Space (RKHS). The non-parametric models consisted of three ML approaches: random forest (RF), support vector machine (SVM), and extreme gradient boosting regression (XGB). Detailed information on each model and the optimisation processes is provided below.

### Ridge regression best linear unbiased prediction (RRBLUP)

The RRBLUP model served as a reference model to compare its performance with other genomic prediction models in wheat breeding. It assumes that all markers contribute equally to the trait, with constant effect variance across markers. Marker effects and variance components were estimated using the restricted maximum likelihood (REML) method, incorporating both phenotypic and marker data [49]. In this study, RRBLUP was implemented using the R package “*rrBLUP*”, specifically through the mixed.solve function (rrBLUP::mixed.solve.). Mathematically, the model can be expressed as:

$$\:y=\mu\:+Zu+{\epsilon\:}$$

Where $\:\mu\:$ is the overall mean, denotes the vector of phenotypic means. The marker matrix is denoted by *Z*, which has dimensions *N×M*. The vector represents random marker effects, which follow a normal distribution with a constant variance, expressed as *u ~ N* (*0*,* I* $\:{\sigma\:}_{u}^{2}$), *i* identity matrix and ε refers to the residual errors, distributed as ε ∼ *N* (0, *I* $\:{\sigma\:}_{e}^{2}$). The solution to the mixed model equations is given by:

$${\text{u}}={{\text{Z}}^{\text{T}}}{\left( {{\text{Z}}{{\text{Z}}^{\text{T}}}+\lambda {\text{I}}} \right)^{ - 1}}{\text{y}}$$

In this equation, *u*, *Z*, and *y* have the same definitions as those above, *I* refers to the identity matrix, and the ridge regression parameter $\:{\uplambda\:}$ is defined as $\:{\uplambda\:}=\frac{{\sigma\:}_{e}^{2}}{{\sigma\:}_{u}^{2}}$ [49, 50].

### Bayesian models

Five Bayesian models were applied: Bayesian Ridge Regression, Bayesian Least Absolute Shrinkage and Selection Operator (Bayes LASSO), Bayes A, Bayes B, and Bayes C. These models differ in how they assign prior distributions to marker effects and variances: Bayesian Ridge Regression uses a normal prior for marker effects, leading to shrinkage similar to the RR-BLUP model. Bayes A uses an inverted chi-squared distribution to estimate marker variances. Bayes B offers a more practical approach for breeding by assuming that not all markers influence the phenotype, utilising a mixture of prior distributions with a substantial probability mass at zero, whereas the remainder follows a Gaussian distribution. Both Bayes C and Bayes LASSO adopt a mixture of prior distributions: Bayes C combines a point mass at zero with a scaled t-distribution, whereas Bayes LASSO applies a double exponential distribution. All Bayesian models were implemented using the “*BGLR*” package in R [51]. The general mixed model framework remains similar as described above:

$$\:y=\mu\:+Zu+\in\:$$

Where the marker effects (*u)*, follows a distribution based on the Bayesian model:

- Bayes A: $\:u\:\sim\:N\left(0{\sigma\:}_{i}^{2}\right)$ allowing each marker its own variance, where marker variance $\:{\sigma\:}_{i}^{2}$ follows an inverted chi-square distribution.
- Bayes B: A mixture model, where with probability π, *ui* = 0, otherwise *ui* ∼ *N* (0, $\:{\sigma\:}^{2}$), assuming only some markers influence the phenotype.
- Bayes C: Similar to Bayes B, but with a scaled-t distribution instead of a Gaussian for non-zero effects.
- Bayesian Ridge Regression (BRR): *u* ∼ *N* (0, $\:{\sigma\:}_{i}^{2}$) applying Gaussian priors to marker effects, similar to RR-BLUP.
- Bayes LASSO: *u ~ Laplace (0*, λ) Assigns a double exponential (Laplace) prior to marker effects, inducing stronger shrinkage for small effects.

The key difference among these models lies in the prior assigned to the marker effects and residual contributions to phenotypic variation. The computational procedures involved 30,000 Monte Carlo Markov chain iterations, with the initial 10,000 iterations as burn-in.

### Reproducing kernel hilbert space (RKHS)

RKHS is a non-parametric method that captures non-linear relationships between markers and phenotypes using kernel functions. The RKHS model is defined as

$$\:y=K\alpha\:+\in\:.$$

where is the vector of phenotypic observations, is the kernel matrix measuring the genetic similarity between individuals, is the vector of random effects associated with the kernel and is the residual error $\propto \sim N\left( {0,\sigma _{ \propto }^{2}K} \right)$, $\in \sim N\left( {0,I\sigma _{ \propto }^{2}} \right)$.

In RKHS, instead of estimating individual marker effects, the focus is on estimating the relationships between individuals in the population using the . *K* was a Gaussian kernel built from the standardized genotype matrix *M* (n × p; n = lines, p = markers) Denote by $\:{x}_{i}$ the i-th row of (the marker vector for line *i*). We computed $d_{{ij}}^{2}=~\left\| {{x_i} - {x_j}} \right\|{~^2}$, and the Gaussian kernel $\:{K}_{ij}=exp\left(-\frac{{d}_{ij}^{2}}{{2h}^{2}}\right)$. The bandwidth $\:h$ was chosen from a small grid around the median-distance heuristic $\:h$ $\:\in\:${0.5,1,2,4}× median ($\:{d}_{ij}^{2}$) by inner cross-validation. The RKHS models were implemented using the BGLR R package [51], following the same 30,000 Monte Carlo Markov chain iterations with the initial 10,000 burn-in. We use RKHS with a Gaussian kernel as implemented in BGLR, a routine choice in crop genomic selection to capture potential non-linear (epistatic) effects. RKHS was fit by supplying *K* in ETA = list(list(K = K, model = “RKHS”)); posterior means were used for prediction.

### Random forest (RF)

Random Forest (RF) is an ensemble learning method that constructs multiple decision trees and aggregates their predictions for increased accuracy. Key steps in RF implementation include:

- Bootstrap sampling: For each tree in the forest, a bootstrap sample is drawn from the training dataset with replacement. This allows for the inclusion of some samples multiple times, while others may be omitted.
- Feature selection: At each node in a tree, the SNP markers (SNP_j_, j = (1,. . ., J)) are evaluated using the mtry parameter to find the subset that minimises the root mean squared error (RMSE). This ensures that the most relevant features are used to split the nodes.
- Tree construction: Trees are grown by recursively splitting nodes until the maximum depth or minimum node size is reached. Predictions for genotype *x*_*i*_ were averaged across all trees in the forest:

$\hat {y}i=\frac{1}{B}\mathop \sum \limits_{{b=1}}^{B} {T_b}\left( {{x_i}} \right)$ where $\hat {y}i$ is the predicted value, $\:{T}_{b}$ is the prediction from the -th tree, and is the number of bootstrap samples.

- Hyperparameter optimization: Key parameters (maxdepth, mtry, ntree) were optimized using randomized grid search cross-validation with the randomForest and caret libraries in R [52, 53]. Hyperparameter tuning was performed to optimise the model for each trait. The ranges included maximum depths (40, 60, 80, 100), features (auto, sqrt), and tree counts (200, 300, 500, and 1000). The optimal parameters were selected based on minimising the Root Mean Square Error (RMSE) for each trait, ensuring improved predictive performance. This approach allowed for fine-tuning of the RF model, providing trait-specific parameter sets that achieved the best predictive accuracy.

### Support vector machine (SVM)

The Support Vector Machine (SVM) was employed to model the relationship between genetic markers and phenotypic traits, via a non-linear kernel to map the predictor space into a high-dimensional feature space. This approach facilitates the study of complex relationships between genotypic and phenotypic data.

The SVM model is represented by the learning function as follows:${{f}}\left( x \right) = wT\phi \left( x \right) + b$

where f(x) is the prediction function, w denotes the weight vector, ϕ(x) represents the non-linear mapping of the input features x into the high-dimensional marker set, and is the bias term. To optimise the model, the SVM minimises the following loss function implicitly used in the “*svm*” function from the e1071 package in R [54], which by default employs a radial basis function (RBF) kernel.

$${\text{min}}\left( {C\mathop \sum \limits_{{i=1}}^{n} L\left( {{e_i}} \right)+~\frac{1}{2}~{{\left\| w \right\|}^2}} \right)$$

Here, is a positive regularization parameter that controls the trade-off between maximizing the margin and minimizing classification errors. The term $\frac{1}{2}~{\left\| w \right\|^2}$ represents the model complexity, and $\:L\left({e}_{i}\right)$ is the loss function for the error $\:{e}_{i}={y}_{i}-f\left({x}_{i}\right)$, where $\:{e}_{i}$ is the deviation of the predicted value from the actual value $\:{y}_{i}$ with the *i*-th training point. SVM aims to find the optimal hyperplane that maximises the margin between different classes while minimising classification errors. The use of kernel non-linear the model to handle nonlinear relationships by transforming the input data into a higher-dimensional space, where linear separation is more effective.

### Extreme gradient boosting (XGB)

The XGB is a boosting algorithm that iteratively improves predictions by minimizing loss through gradient descent. It constructs a series of decision trees, with each tree correcting the errors of the previous ones. XGB is highly efficient, scalable, and well-suited for handling large datasets while delivering accurate predictions through iterative refinement. It predicts phenotypic traits by employing an ensemble of decision trees in a boosting framework. XGB uses a gradient-boosting to iteratively improve model predictions by minimising the loss function through gradient descent. The model is defined by the following objective function:

$$L\left( {y,\hat {y}} \right)=\mathop \sum \limits_{{i=1}}^{n} L\left( {{y_i},{{\hat {y}}_i}} \right)+\mathop \sum \limits_{{K=1}}^{K} {\text{\varvec{\Omega}}}\left( {{f_K}} \right)$$

where $L\left( {{y_i},{{\hat {y}}_i}} \right)$ is the loss function measuring the difference between the predicted value $\:{\hat{y}}_{i}$ is obtained by adding the output of *K* regression trees applied to the vector ${\hat {y}_i}=\mathop \sum \limits_{{K=1}}^{K} {f_k}$(SNPs of *i*), K= number of trees, $\:{f}_{K}$ = the *k*-th CART regression tree with leaf weights $\:{w}_{kj}^{\:}$ and the true value $\:{y}_{i}$ and Ω$\:\left({f}_{K}\right)$ represents the regularization term that penalizes model complexity: Ω$\:\left({f}_{K}\right)=\gamma\:\#leaves+\frac{\lambda\:}{2}{\sum\:}_{j}{w}_{kj}^{2}$. The analysis was performed using “xgboost” package in R [55]. The model by minimum RMSE in cross-validation. For regression tasks, the squared error loss function is employed as.

$$L\left( {y,\hat {y}} \right)=\frac{1}{n}\mathop \sum \limits_{{i=1}}^{n} {\left( {{y_i}~ - {{\hat {y}}_i}} \right)^2}$$

### Prediction accuracy and cross-validation scheme

Prediction accuracy was assessed using a K-fold cross-validation approach (KFCV), where the dataset was divided into a training set (TS) comprising 80% of the data and a validation set (VS) consisting of the remaining 20% within each environmental condition. Model performance was evaluated over 150 iterations, with prediction accuracy quantified as the Pearson correlation coefficient between observed phenotypes and genomic estimated breeding values (GEBVs) averaged over all iterations.

Additionally, a leave-one-out cross-validation (LOOCV) approach was employed to further validate model robustness. In this approach, each data point is sequentially used as a test set, while the remaining data formed the training set, maximizing the use of available data for validation. Predictive accuracy was determined through correlation analysis between predicted GEBVs and observed phenotypes. Both K-fold cross-validation and leave-one-out cross-validation procedures were implemented in R software.

The study analyzed well-watered (WW) and water-stressed (WS) conditions over two years (2021 and 2022) with data representing the mean genotype values across both years for each treatment. Cross-validation was also performed on the combined mean data of both water regimes, referred to as the combined water regime (CWR) were also done in R software. This involved three configurations: (1) training on WW data and testing on WS data; (2) the reverse configuration, training on WS data and testing on WW data and (3) training on CWR data with separate predictions for WS and WW.

## Results

### Phenotypic and climatic variables

Trait evaluation was conducted under WS and WW conditions for two years (2021 and 2022). As shown in Table 1, temperatures (average, maximum, and minimum) consistently increased in 2022 compared to 2021. The distribution of all traits, including grain yield (GY), across environments is provided in Supplementary Fig. 1. Table 2 presents the variations in mean performance, variance components, and heritability of different traits under WS and WW conditions. Heritability estimates (H²) ranged from 0.20 (GY under WS) to 0.87 (TKW under CWR). Within trails the H^2^ ranged from 0.20 (GY) to 0.72 (NGS) in WS, from 0.34 (GY) to 0.85 (GP) under WW, and from 0.48 (GY) to 0.87 (TKW) under CWR. Under WS, grain yield (GY) had a mean of 6.39 t/ha with low heritability (H² = 0.20), whereas under WW and CWR, GY increased to 8.68 t/ha (H² = 0.34) and 7.53 t/ha (H² = 0.48), respectively. showed moderate to high heritability across all regimes, increasing from 0.69 (WS) to 0.74 (WW) and high at 0.87 under CWR. Similarly, both NSm^2^ and NGS had higher mean values and heritability under WW and CWR compared with WS. PH recorded mean values of 79.46 cm (H² = 0.49) under WS, 88.74 cm (H² = 0.69) under WW, and 84.09 cm (H² = 0.77) under CWR. GP averaged 15.67% (H² = 0.60) in WS, 13.74% (H² = 0.85) in WW, and 14.71% (H² = 0.83) in CWR.

**Table 2:** Genetic variability parameters of different traits under WS and WW conditions

Trail	Trait	Mean ± SE	Range	GV	PV	EV	GCV	PCV	ECV	H^2^
**WS**	GY	6.39 ± 0.06	4.22–8.23	0.10	0.50	0.24	5.08	11.35	7.76	0.20
**WS**	TKW	37.77 ± 0.27	30.40-50.14	9.89	14.33	2.99	8.40	10.11	4.62	0.69
**WS**	NSm^2^	356.70 ± 4.05	231.35-525.73	855	2719.32	1159.58	8.31	14.81	9.67	0.32
**WS**	NGS	46.57 ± 0.41	27.63–61.94	20.06	28.16	5.02	9.54	11.30	4.77	0.72
**WS**	PH	79.46 ± 0.33	66.89–92.48	9.88	20.50	8.76	3.96	5.70	3.73	0.49
**WS**	GP	15.67 ± 0.06	13.77–18.97	0.33	0.56	0.10	3.65	4.75	1.98	0.60
**WW**	GY	8.68 ± 0.08	6.08–11.17	0.32	0.95	0.39	6.55	11.23	7.22	0.34
**WW**	TKW	44.35 ± 0.34	33.88–57.02	13.4	18.24	2.22	8.28	9.66	3.37	0.74
**WW**	NSm^2^	406.92 ± 4.81	268.27-593.72	1559.86	4009.47	1426.73	9.71	15.56	9.28	0.39
**WW**	NGS	49.15 ± 0.44	29.68–65.29	21.47	29.29	4.90	9.36	10.93	4.47	0.74
**WW**	PH	88.74 ± 0.31	78.29–98.85	12.31	18.03	3.55	3.96	4.79	2.13	0.69
**WW**	GP	13.74 ± 0.06	11.80-16.17	0.51	0.60	0.05	5.16	5.62	1.49	0.85
**CWR**	GY	7.53 ± 0.06	5.15–9.19	0.23	0.48	0.16	6.44	9.32	5.31	0.48
**CWR**	TKW	41.06 ± 0.29	32.15–51.62	12.78	14.82	1.30	8.75	9.42	2.79	0.87
**CWR**	NSm^2^	381.99 ± 3.85	258.33-522.97	1579.59	2567.66	645.41	10.47	13.34	6.69	0.62
**CWR**	NGS	47.83 ± 0.40	30.56–63.62	21.01	24.91	2.48	9.50	10.35	3.26	0.85
**CWR**	PH	84.09 ± 0.29	73.06–95.67	12.38	16.15	3.08	4.19	4.78	2.09	0.77
**CWR**	GP	14.71 ± 0.06	12.79–17.40	0.41	0.49	0.04	4.33	4.75	1.26	0.83

WS- Water Stress; WW- Well Watered; CWR - Combined Water Regimes(WS & WW); GY- Grain Yield (t/ha); TKW- Thousand Kernel Weight (g); NSm^2^- Number of spikes per m^2^; NGS- Number of grains per Spike; PH- Plant Height (cm); GP- Grain Protien (%); GV-Genetic Variance; PV- Phenotypic Variance; EV- Environmental Variance; GCV- Genetic Coefficient of Variance; PCV- Phenotypic Coefficient of Variance; ECV- Environmental Coefficient of Variance Trail: Growing condition (WS = Water Stress, WW = Well-Watered; Mean ± SE: Mean value with standard error; Range: Minimum to maximum observed values; H² (Heritability): Broad sense heritability

The GCV across traits ranged from 3.65% (GP under WS) to 10.47% (NSm² under CWR), indicating varying degrees of genetic influence. PCV values were consistently higher than GCV across all traits, ranging from 4.75% (GP under WS and CWR) to 15.56% (NSm² under WW), emphasizing the significant role of environmental factors in overall trait variation. This effect was particularly evident in NSm^2^ and GY. The ECV values ranged from 1.26% (GP under CWR) to 9.67% (NSm² under WS), further underscoring the influence of environmental conditions (Table 2).

A comparison between WW and WS conditions revealed that WW conditions mitigate the environmental variability for several traits. For example, the ECV for GY decreased from 7.76% under WS to 7.22% under WW, indicating that irrigation reduced environmental variability. TKW exhibited moderate heritability under both WS (H^2^ = 0.69) and WW conditions (H^2^ = 0.74), along with relatively low ECV values (4.62% under WS and 3.37% under WW) compared to traits such as GY, indicating that TKW is less affected by environmental fluctuations, making it a more stable trait. However, the ECV of NGS (4.77% under WS and 4.47% under WW) reflected the environmental sensitivity of this trait, although the environmental impact was relatively consistent across different water regimes. Similarly, traits such as PH and NSm^2^ exhibited lower ECV under WW conditions, suggesting that adequate water supply helps to buffer against environmental fluctuations, thereby enhancing the relative impact of genetic factors.

### Trait correlations

**Fig. 1:**
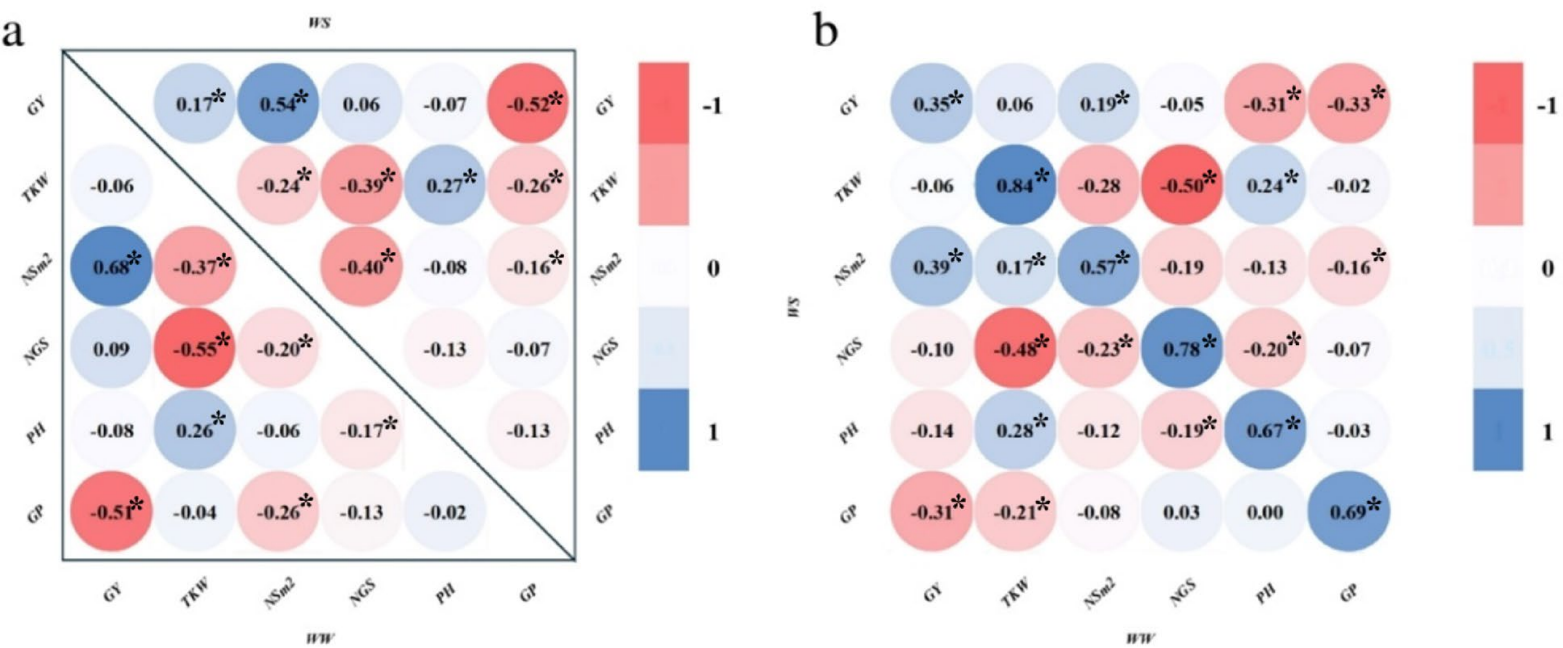
Pearson’s correlations of traits under different water conditions: (A) Within-condition correlations under WS (lower left) and WW (upper right); (B) Cross-condition correlations. GY- Grain Yield (t/ha); TKW- Thousand Kernel Weight (g); NSm^2^- Number of spikes per m^2^; NGS- Number of grains per Spike; PH- Plant Height (cm); GP- Grain Protein (%)

Under WW a significant negative correlation was observed between TKW and NGS (*r* = -0.55), while NSm^2^ demonstrated a positive correlation with GY (*r* = 0.68) (Fig. 1a). Under WW and WS conditions, a notable negative correlation was observed between GY and GP (*r* = -0.52, *r* = -0.51 respectively). The correlation between GY and NSm^2^ was positive under WS and WW conditions (*r* = 0.54, 0.68), highlighting the critical role of spike density in yield formation. Additionally, PH was positively correlated with GY (*r* = 0.72) under WS conditions.

### Cross-condition correlations

Cross-condition correlation analysis revealed significant correlations among all traits across different water availability scenarios, highlighting their relative stability and variability to environmental variation. GP exhibited moderate positive correlations between WW and WS conditions (*r* = 0.69), as did in NSm^2^ (*r* = 0.57) and PH (*r* = 0.67) (Fig. 1b), indicating the stability of these traits across conditions. However, TKW demonstrated strong correlation across conditions (*r* = 0.84), suggesting that genetic and environmental interactions could influence the consistency of this trait. Conversely, GY showed a low positive correlation (*r* = 0.35) between the conditions, highlighting its sensitivity to environmental variation. Notably, a negative correlation was found between NGS under WS conditions and TKW under WW conditions (*r* = -0.48), suggesting that an increase in grain numbers under drought stress may not translate into kernel weight under irrigation. Additionally, GP under WS conditions negatively correlated with GY (-0.31), and TKW (-0.21) in WW conditions.

### Cross-validation analysis of genomic selection models on the training set

#### K fold cross-validation (KFCV)

This study comprehensively evaluated model performance across various conditions and traits using 150 iterations of k-fold cross-validation with an 80:20 data split. The mean *r*_*MG*_ across traits and conditions was highest for RR-BLUP and RF (0.66), while SVM and XGB achieved mean *r*_*MG*_ values of 0.37 and 0.53, respectively. Overall, RR-BLUP achieved the highest average *r*_*MG*_ across traits, with the highest values observed under WS conditions (mean *r*_*MG*_ = 0.72), followed by CWR (0.65) and WW (0.64). Under WS, RR-BLUP yielded particularly high *r*_*MG*_ values for GY (0.75), TKW (0.73), and PH (0.71). Similarly, under CWR, high *r*_*MG*_ values were recorded for PH (0.71), TKW (0.70), and GP (0.65). For GY, RR-BLUP achieved *r*_*MG*_ values of 0.75 (WS), 0.72 (WW) and 0.65 (CWR) (Table 3). The RF model performed strongly, especially for GY and TKW, with *r*_*MG*_ values improving under WW, achieving 0.74 (WS) and 0.81 (WW) for GY, and 0.67 (WS) and 0.73 (WW) for TKW. The mean *r*_*MG*_ of all traits for RF and XGB models was alsonotable, with values of WS = 0.67 and 0.55, WW = 0.67 and 0.52, and CWR of 0.66 and 0.53, respectively. XGB performed especially stable for GY (*r*_*MG*_ ~0.60) and GP (*r*_*MG*_ ~0.48) across all regimes.

Bayesian models displayed *r*_*MG*_ values for GY of 0.23 to 0.25 under CWR, 0.47 to 0.49 under WS, and negative under WW, with all traits lowest under WW. Although lower than other models, CWR *r*_*MG*_ values were consistently below WS. SVM exhibited moderate prediction accuracy, with mean *r*_*MG*_ for across all traits and conditions was 0.38 (WS), 0.34 (WW), and 0.40 (CWR). Performance varied across traits, with the best results observed for TKW, PH, and GP under WS condition; TKW and PH under WW condition; and GY, TKW, PH, and GP under CWR condition. Bayesian models and RKHS typically had lower *r*_*MG*_ values, acting as benchmarks rather than top performers.

**Table 3:** Prediction performance of different GS models under different water conditions using K-Fold cross-validation (KFCV)

Trail	Trait	RR-Blup		Bayes A		Bayes B		Bayes C		BRR	
		*r*_MP_ ± SD	*r*_MG_	*r*_MP_ ± SD	*r*_MG_	*r*_MP_ ± SD	*r*_MG_	*r*_MP_ ± SD	*r*_MG_	*r*_MP_ ± SD	*r*_MG_
**WS**	**GY**	0.34 ± 0.12	0.75	0.21 ± 0.02	0.47	0.21 ± 0.01	0.47	0.22 ± 0.01	0.49	0.21 ± 0.01	0.47
	**TKW**	0.61 ± 0.11	0.73	0.13 ± 0.01	0.16	0.13 ± 0.01	0.16	0.14 ± 0.01	0.17	0.14 ± 0.01	0.17
	**NSm2**	0.39 ± 0.14	0.68	0.06 ± 0.02	0.11	0.07 ± 0.02	0.12	0.07 ± 0.02	0.12	0.08 ± 0.02	0.14
	**NGS**	0.54 ± 0.12	0.63	0.04 ± 0.01	0.05	0.04 ± 0.01	0.05	0.05 ± 0.01	0.06	0.04 ± 0.01	0.05
	**PH**	0.50 ± 0.11	0.71	-0.33 ± 0.02	-0.47	-0.31 ± 0.02	-0.44	-0.29 ± 0.02	-0.41	-0.30 ± 0.02	-0.43
	**GP**	0.53 ± 0.14	0.68	-0.12 ± 0.01	-0.15	-0.13 ± 0.01	-0.17	-0.13 ± 0.01	-0.17	-0.13 ± 0.01	-0.17
	**Mean**	0.48	0.7	0.00	0.03	0.00	0.03	0.01	0.04	0.01	0.04
**WW**	**GY**	0.44 ± 0.13	0.72	-0.15 ± 0.02	-0.26	-0.17 ± 0.02	-0.29	-0.18 ± 0.02	-0.31	-0.18 ± 0.02	-0.31
	**TKW**	0.61 ± 0.11	0.74	-0.16 ± 0.02	-0.19	-0.14 ± 0.02	-0.16	-0.13 ± 0.02	-0.15	-0.14 ± 0.02	-0.16
	**NSm2**	0.39 ± 0.13	0.61	-0.17 ± 0.01	-0.27	-0.17 ± 0.01	-0.27	-0.17 ± 0.01	-0.27	-0.17 ± 0.01	-0.27
	**NGS**	0.52 ± 0.12	0.57	0.15 ± 0.02	0.17	0.17 ± 0.02	0.2	0.18 ± 0.02	0.21	0.18 ± 0.02	0.21
	**PH**	0.59 ± 0.12	0.55	-0.21 ± 0.01	-0.25	-0.2 ± 0.02	-0.24	-0.2 ± 0.01	-0.24	-0.2 ± 0.02	-0.24
	**GP**	0.62 ± 0.09	0.67	-0.36 ± 0.02	-0.39	-0.37 ± 0.01	-0.4	-0.38 ± 0.02	-0.41	-0.38 ± 0.01	-0.41
	**Mean**	0.52	0.64	-0.15	-0.20	-0.15	-0.19	-0.15	-0.20	-0.15	-0.20
**CWR**	**GY**	0.45 ± 0.13	0.65	0.16 ± 0.02	0.23	0.17 ± 0.02	0.25	0.17 ± 0.02	0.25	0.17 ± 0.02	0.25
	**TKW**	0.66 ± 0.1	0.7	-0.13 ± 0.01	-0.14	-0.14 ± 0.01	-0.15	-0.14 ± 0.01	-0.15	-0.14 ± 0.01	-0.15
	**NSm2**	0.46 ± 0.13	0.57	0.08 ± 0.03	0.10	0.06 ± 0.02	0.08	0.05 ± 0.02	0.06	0.05 ± 0.02	0.06
	**NGS**	0.56 ± 0.12	0.60	0.07 ± 0.01	0.08	0.07 ± 0.01	0.08	0.07 ± 0.01	0.08	0.07 ± 0.01	0.08
	**PH**	0.63 ± 0.1	0.71	0.06 ± 0.02	0.07	0.04 ± 0.02	0.05	0.02 ± 0.02	0.02	0.04 ± 0.02	0.05
	**GP**	0.59 ± 0.11	0.65	-0.31 ± 0.01	-0.34	-0.31 ± 0.01	-0.34	-0.31 ± 0.01	-0.34	-0.31 ± 0.01	-0.34
	**Mean**	0.55	0.65	-0.01	0.00	-0.02	-0.01	-0.02	-0.01	-0.02	-0.01
**Grand mean**		0.52	0.66	-0.05	-0.06	-0.06	-0.06	-0.05	-0.06	-0.05	-0.06

Trail	Trait	LASSO		RKHS		Random Forest		SVM		Extreme Gradient Boosting	
		*r*_MP_ ± SD	*r*_MG_	*r*_MP_ ± SD	*r*_MG_	*r*_MP_ ± SD	*r*_MG_	*r*_MP_ ± SD	*r*_MG_	*r*_MP_ ± SD	*r*_MG_
**WS**	**GY**	0.21 ± 0.01	0.47	0.24 ± 0.01	0.54	0.33 ± 0.13	0.74	0.16 ± 0.13	0.36	0.28 ± 0.14	0.63
	**TKW**	0.13 ± 0.01	0.16	0.13 ± 0.01	0.16	0.56 ± 0.09	0.67	0.39 ± 0.14	0.47	0.43 ± 0.15	0.52
	**NSm** ^**2**^	0.05 ± 0.04	0.09	0.10 ± 0.01	0.18	0.40 ± 0.13	0.71	0.22 ± 0.15	0.39	0.35 ± 0.15	0.62
	**NGS**	0.04 ± 0.02	0.05	0.05 ± 0.01	0.06	0.56 ± 0.12	0.66	0.08 ± 0.18	0.09	0.42 ± 0.15	0.49
	**PH**	-0.32 ± 0.02	-0.46	-0.31 ± 0.01	-0.44	0.46 ± 0.12	0.66	0.33 ± 0.13	0.47	0.37 ± 0.12	0.53
	**GP**	-0.13 ± 0.02	-0.17	-0.12 ± 0.01	-0.15	0.46 ± 0.14	0.59	0.38 ± 0.17	0.49	0.37 ± 0.17	0.48
	**Mean**	0.00	0.02	0.02	0.06	0.46	0.67	0.26	0.38	0.37	0.55
**WW**	**GY**	-0.18 ± 0.03	-0.31	-0.19 ± 0.02	-0.33	0.47 ± 0.12	0.81	0.27 ± 0.15	0.46	0.35 ± 0.15	0.6
	**TKW**	-0.15 ± 0.03	-0.17	-0.13 ± 0.02	-0.15	0.63 ± 0.09	0.73	0.45 ± 0.14	0.52	0.35 ± 0.15	0.41
	**NSm** ^**2**^	-0.17 ± 0.03	-0.27	-0.17 ± 0.01	-0.27	0.39 ± 0.12	0.62	0.19 ± 0.17	0.3	0.29 ± 0.17	0.46
	**NGS**	0.16 ± 0.03	0.19	0.17 ± 0.01	0.2	0.51 ± 0.11	0.59	0.14 ± 0.2	0.16	0.43 ± 0.15	0.5
	**PH**	-0.21 ± 0.02	-0.25	-0.19 ± 0.02	-0.23	0.52 ± 0.12	0.63	0.38 ± 0.16	0.46	0.55 ± 0.11	0.66
	**GP**	-0.38 ± 0.02	-0.41	-0.37 ± 0.01	-0.4	0.57 ± 0.09	0.62	0.13 ± 0.17	0.14	0.43 ± 0.14	0.47
	**Mean**	-0.16	-0.2	-0.15	-0.2	0.52	0.67	0.26	0.34	0.4	0.52
**CWR**	**GY**	0.17 ± 0.02	0.25	0.18 ± 0.02	0.26	0.49 ± 0.12	0.71	0.31 ± 0.14	0.45	0.43 ± 0.13	0.62
	**TKW**	-0.13 ± 0.01	-0.14	-0.14 ± 0.01	-0.15	0.62 ± 0.1	0.66	0.46 ± 0.13	0.49	0.52 ± 0.13	0.56
	**NSm** ^**2**^	0.1 ± 0.04	0.13	0.04 ± 0.02	0.05	0.47 ± 0.12	0.6	0.26 ± 0.14	0.33	0.37 ± 0.15	0.47
	**NGS**	0.07 ± 0.01	0.08	0.06 ± 0.01	0.07	0.56 ± 0.11	0.61	0.14 ± 0.19	0.15	0.44 ± 0.15	0.48
	**PH**	0.05 ± 0.02	0.06	0.02 ± 0.02	0.02	0.65 ± 0.09	0.74	0.41 ± 0.15	0.47	0.52 ± 0.11	0.59
	**GP**	-0.31 ± 0.01	-0.34	-0.33 ± 0.01	-0.36	0.54 ± 0.1	0.59	0.44 ± 0.14	0.48	0.44 ± 0.15	0.48
	**Mean**	-0.01	0.01	-0.03	-0.02	0.56	0.65	0.34	0.4	0.45	0.53
**Grand mean**		-0.06	-0.06	-0.05	-0.05	0.51	0.66	0.29	0.37	0.41	0.53

WS – Water Stress, WW – Well-Watered, CWR – Combined Water Regimes - WS & WW; GY– Grain Yield (t/ha), TKW – Thousand Kernel Weight (g); NSm^2^ – Number of Spikes per m²; NGS – Number of Grains per Spike; PH – Plant height (cm); GP – Grain Protein Content (%); RR-Blup -Ridge Regression Best Linear Unbiased Prediction; Bayes A – Bayesian Regression Model A; Bayes B – Bayesian Regression Model B; Bayes C – Bayesian Regression Model C; BRR – Bayesian Ridge Regression; LASSO – Least Absolute Shrinkage and Selection Operator; RKHS – Reproducing Kernel Hilbert Space; RF – Random Forest; SVM – Support Vector Machine; XGB – Extreme Gradient Boosting; *r*_*MP*_ – Predictive Ability; *r*_*MG*_ –Prediction Accuracy; SD – Standard Deviation.

### Leave one out cross validation (LOOCV)

Due to suboptimal performance and high computational demands, Bayesian and RKHS models were excluded from LOOCV analysis, with the focus on regression-based and ML models. RR-BLUP achieved the highest mean *r*_*MG*_ across traits and conditions (0.68), followed by RF (0.66) and XGB (0.56), while SVM showed the lowest (0.36) (Table 4). Among these conditions, WS yielded the highest average *r*_*MG*_ (0.70 for RR-BLUP, 0.68 for RF), followed by CWR (0.65 RR-BLUP, 0.64 RF), and WW (0.68 for both RR-BLUP and RF), particularly for GY, TKW, PH, and GP, with *r*_*MG*_ values exceeding 0.65 in most cases. For example, GY was predicted with high accuracy by RR-BLUP (*r*_*MG*_ = 0.69–0.75) and RF (0.72–0.78) across WS and WW. TKW and PH showed better accuracy, especially under CWR (TKW: 0.69 RR-BLUP, 0.68 RF; PH: 0.72 RR-BLUP, 0.73 RF). GP prediction was particularly robust under WS and CWR, with *r*_*MG*_ values reaching 0.70 for RR-BLUP and 0.63 for RF (Table 4).

XGB showed moderate but stable prediction ability across traits and conditions, with *r*_*MG*_ values ranging from 0.38 to 0.71, and was particularly effective for TKW, PH, and GY under WW and CWR. While not as higher as RR-BLUP or RF, XGB consistently outperformed SVM and demonstrated lower RMSE than SVM for nearly all traits. SVM exhibited the lowest predictive accuracy and highest RMSE across all conditions, with trait-wise *r*_*MG*_ values rarely exceeding 0.50 (Table 4). The model was particularly weak for NSm² and NGS, but showed modest accuracy for TKW in WW (*r*_*MG*_ = 0.52) and GP in WS (*r*_*MG*_ = 0.55). However, its performance was inconsistent across conditions, with declines in WW and CWR for several traits compared to other models. For example, SVM predicted GP relatively well under WS (*r*_*MG*_ = 0.55), yet its performance dropped sharply under WW (*r*_*MG*_ = 0.17), while RR-BLUP and RF maintained consistently high accuracy across all conditions.

**Table 4:** Prediction performance of different GS models under different water conditions using leave-one-out cross-validation (LOOCV)

Trail	Trait	RR Blup			RF			SVM			XGB		
		*r*_*MP*_	*r*_*MG*_	RMSE	*r*_*MP*_	*r*_*MG*_	RMSE	*r*_*MP*_	*r*_*MG*_	RMSE	*r*_*MP*_	*r*_*MG*_	RMSE
WS	GY	0.31	0.69	0.73	0.32	0.72	0.73	0.13	0.30	0.95	0.30	0.67	0.79
	TKW	0.62	0.75	2.84	0.55	0.66	3.12	0.38	0.46	3.89	0.38	0.46	3.57
	NSm^2^	0.39	0.69	49.77	0.40	0.71	49.55	0.21	0.37	69.86	0.40	0.71	52.19
	NGS	0.52	0.62	4.69	0.54	0.64	4.70	0.05	0.06	5.82	0.42	0.50	5.45
	PH	0.51	0.73	3.76	0.50	0.72	3.80	0.30	0.43	5.42	0.35	0.50	4.45
	GP	0.54	0.70	0.65	0.49	0.63	0.68	0.42	0.55	0.78	0.40	0.52	0.74
	WS Mean	0.48	0.70	10.40	0.47	0.68	10.43	0.25	0.36	14.45	0.38	0.56	11.20
WW	GY	0.43	0.75	0.92	0.46	0.78	0.91	0.26	0.44	1.23	0.31	0.53	1.06
	TKW	0.62	0.72	3.60	0.62	0.72	3.71	0.44	0.52	5.07	0.60	0.69	3.73
	NSm^2^	0.39	0.63	59.07	0.38	0.61	59.25	0.17	0.27	84.33	0.24	0.38	68.75
	NGS	0.51	0.60	5.09	0.50	0.59	5.20	0.13	0.15	7.30	0.51	0.59	5.25
	PH	0.60	0.72	3.31	0.64	0.76	3.26	0.36	0.43	5.16	0.58	0.70	3.51
	GP	0.63	0.68	0.60	0.56	0.60	0.65	0.16	0.17	0.96	0.45	0.49	0.70
	WW Mean	0.53	0.68	12.10	0.53	0.68	12.16	0.25	0.33	17.34	0.45	0.56	13.83
CWR	GY	0.45	0.66	0.66	0.48	0.69	0.65	0.29	0.42	0.87	0.44	0.63	0.71
	TKW	0.65	0.69	3.00	0.63	0.68	3.20	0.45	0.48	4.18	0.60	0.65	3.17
	NSm^2^	0.47	0.6	46.07	0.45	0.57	46.92	0.24	0.31	65.85	0.35	0.44	52.54
	NGS	0.55	0.59	4.53	0.55	0.59	4.61	0.16	0.18	6.12	0.40	0.43	5.32
	PH	0.63	0.72	3.01	0.64	0.73	3.05	0.39	0.44	4.74	0.55	0.63	3.44
	GP	0.60	0.66	0.56	0.51	0.56	0.62	0.47	0.52	0.70	0.49	0.54	0.64
	CWR Mean	0.56	0.65	9.64	0.54	0.64	9.84	0.33	0.39	13.74	0.47	0.55	10.97
	Grand Mean	0.52	0.68	10.71	0.51	0.66	10.81	0.28	0.36	15.18	0.43	0.56	12.00

WS (Water Stress), WW (Well-Watered), CWR (Combined Water Regimes - WS & WW); GY– Grain Yield (t/ha), TKW – Thousand Kernel Weight (g); NSm^2^ – Number of Spikes per m²; NGS – Number of Grains per Spike; PH – Plant height (cm); GP – Grain Protein Content (%); RR-Blup -Ridge Regression Best Linear Unbiased Prediction; RF-Random Forest; SVM- Support Vector Machine; XGB- Extreme Gradient Boosting; *r*_*MP*_ - Predictive Ability; *r*_*MG*_ -Prediction Accuracy; RMSE- Root Mean Square Error

### Comparison of cross-validation methods

**Fig. 2:**
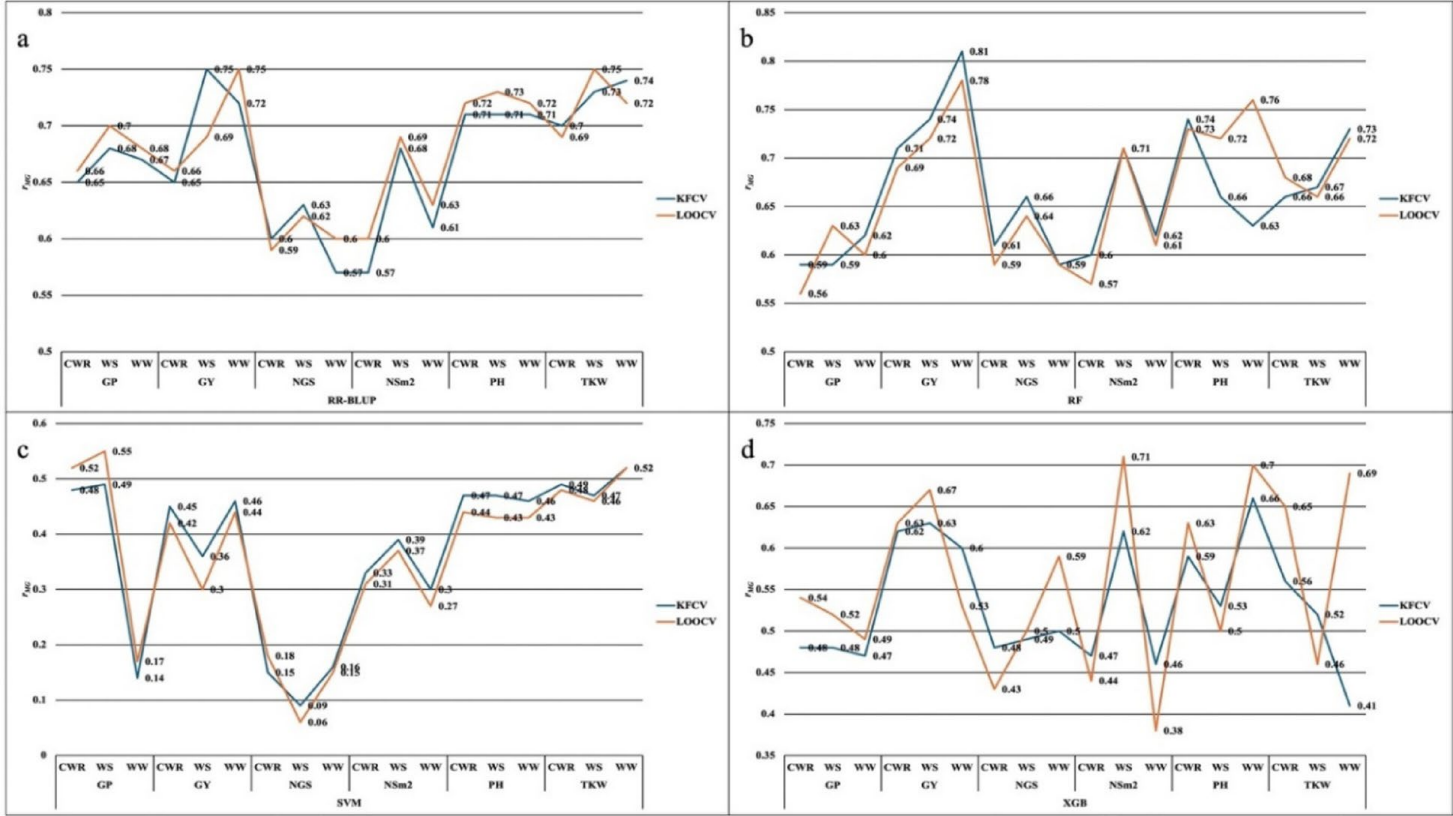
Comparison of GS model prediction performance under water conditions using KFCV and LOOCV methods

A comparison of the *r*_*MG*_ between KFCV and LOOCV across different models and environmental conditions (WS, WW, and CWR) revealed largely consistent results across most models and environmental conditions, with only minor variations for many trait–model combinations (Fig. 2). In WS conditions, k-fold *r*_*MG*_ ranged from 0.09 (SVM) to 0.75 (RR-BLUP). LOOCV accuracies in WS conditions ranged from 0.06 (SVM) to 0.75 (RR-BLUP) (Fig. 2, Supplementary Table 1). RR-BLUP and RF remained the most consistent and high-performing models, with grand mean *r*_*MG*_ values of 0.68 and 0.66 in LOOCV, closely matching their KFCV means (0.67 and 0.66, respectively). Across environments, WS conditions consistently showed the highest *r*_*MG*_ values, with RR-BLUP and RF maintaining nearly identical accuracy in KFCV (0.70 and 0.67) and LOOCV (both ~ 0.70). WW and CWR conditions also showed minimal variation between two cross validation methods, with RR-BLUP and RF *r*_*MG*_ values differing by less than ± 0.02 in most cases. For example, TKW under WW showed stable accuracy for RR-BLUP (0.74 to 0.72) and RF (0.73 to 0.72), indicating that both validation methods yield similar conclusions for stable traits.

A few traits, however, showed notable trait- and model-specific differences. For instance, GP under WS improved slightly in LOOCV for both RR-BLUP (0.68 to 0.70) and RF (0.59 to 0.63). Conversely, GY in WS showed a minor decline for RR-BLUP (0.75 to 0.69), while XGB’s prediction of TKW especially under WW improved from 0.41 (KFCV) to 0.69 (LOOCV), also same trend and slight improvements were observed in WS and CWR conditions. PH in WW also improved with RF (0.63 to 0.76), indicating better generalization in some physiological traits under LOOCV.

WS (Water Stress), WW (Well-Watered), CWR (Combined Water Regimes - WS & WW); GY– Grain Yield (t/ha), TKW – Thousand Kernel Weight (g); NSm^2^ – Number of Spikes per m²; NGS – Number of Grains per Spike; PH – Plant height (cm); GP – Grain Protein Content (%); (a) RR-Blup -Ridge Regression Best Linear Unbiased Prediction; (b) RF-Random Forest; (c) SVM- Support Vector Machine; d)XGB- Extreme Gradient Boosting; *r*_*MG*_ -Prediction Accuracy.

### Cross-condition training and validation analysis

Cross-condition training and validation analysis revealed distinct trends in model performance, especially between models trained in WW conditions and validated in WS conditions, and vice versa. Many models showed *r*_*MG*_ values greater than 1, especially when trained on CWR and tested on individual conditions. This was largely due to high *r*_*MP*_ values, which remained elevated even when H^2^ was low. Because of this significant influence, *r*_*MP*_ was used as a convenient metric for evaluating statistical predictive accuracy. The mean *r*_*MP*_ for RR BLUP was 0.64, while the Bayesian and RKHS models ranged from 0.44 to 0.49. ML models such as SVM, RF, and XGB demonstrated similar performance to RR BLUP, with *r*_*MP*_ of 0.63, 0.65, and 0.65, respectively (Table 5), when trained in WW and validated on WS data. RR BLUP showed superior predictive performance across various traits: GY (*r*_*MP*_ 0.34, RMSE 0.76 t/ha), TKW (*r*_*MP*_ 0.84, RMSE 2.1 g), NSm² (*r*_*MP*_ 0.56, RMSE 44.9 spikes/m²), NGS (*r*_*MP*_ 0.77, RMSE 3.51 grains/spike), PH (*r*_*MP*_ 0.67, RMSE 3.27 cm), and GP (*r*_*MP*_ 0.68, RMSE 0.57%). Bayesian models exhibited lower performance with higher RMSE values, whereas RKHS models performed comparably to RR BLUP.

ML models maintained stable performance compared to traditional models, achieving *r*_*MP*_ values for GY between 0.32 and 0.37 (RMSE ~ 2.5 t/ha), TKW 0.82 to 0.85 (RMSE ~ 7 g), NSm² 0.56 to 0.58 (RMSE 64 to 75 spikes/m²), NGS 0.73 to 0.79 (RMSE ~ 4.5 grains/spike), PH 0.67 to 0.68 (RMSE ~ 9.8 cm), and GP 0.64 to 0.69 (RMSE ~ 2%) (Table 5). When models were trained on WS data and tested on WW data, RR BLUP continued to outperform others: GY (*r*_*MP*_ 0.42, RMSE 0.93 t/ha), TKW (*r*_*MP*_ 0.85, RMSE 2.52 g), NSm² (*r*_*MP*_ 0.57, RMSE 54.12 spikes/m²), NGS (*r*_*MP*_ 0.70, RMSE 4.32 grains/spike), PH (*r*_*MP*_ 0.72, RMSE 2.91 cm), and GP (*r*_*MP*_ 0.70, RMSE 0.55%) (Table 5). Bayesian models generally showed lower performance, but RKHS models were closer to RR BLUP accuracies.

Training on the CWR dataset and prediction for both WW and WS conditions showed consistently high *r*_*MP*_ when tested on WW trials than tested on WS. RR BLUP achieved a mean *r*_*MP*_of 0.81 (WS) and 0.85 (WW), while Bayesian and kernel models ranged from 0.63 to 0.72 (WS) and 0.67 to 0.72 (WW). ML models achieved an *r*_*MP*_ of 0.84 to 0.89 (WS) and 0.87 to 0.92 (WW) (Table 5). Traditional models performed better under irrigated conditions, likely due to stable growing environments. For example, RR BLUP’s *r*_*MP*_ for GY was 0.73 (RMSE 2.43 t/ha) in WW, compared to 0.59 (RMSE 0.62 t/ha) in WS. Bayesian models had moderate predictive power with higher RMSEs and were less effective under rainfed conditions.

ML models consistently outperformed traditional models under all conditions, with XGB emerging as the most robust model. XGB achieved the highest accuracy for GY in WS (*r*_*MP*_ 0.76, RMSE 1.26 t/ha) and WW (*r*_*MP*_ 0.87, RMSE 1.26 t/ha), demonstrating superior ability to manage accuracy and error. It excelled in GP predictions with an *r*_*MP*_ of 0.92 in both WS and WW conditions and low RMSE values (1.01%) (Table 5). The RF model performed well for traits such as TKW and NGS, showing substantial improvements in WW conditions (Table 5).

**Table 5:** Cross-conditions prediction performance of different GS models

	Traits	RR BLUP			Bayes A			Bayes B			Bayes C			BRR		
		*r*_*MP*_	*r*_*MG*_	RMSE	*r*_*MP*_	*r*_*MG*_	RMSE	*r*_*MP*_	*r*_*MG*_	RMSE	*r*_*MP*_	*r*_*MG*_	RMSE	*r*_*MP*_	*r*_*MG*_	RMSE
**Training WW and Testing WS**	**GY**	0.34	0.76	0.8	0.27	0.6	2.41	0.25	0.56	8.68	0.23	0.51	2.41	0.23	0.51	2.41
	**TKW**	0.84	1.01	2.1	0.66	0.79	7.2	0.63	0.76	37.99	0.58	0.7	7.33	0.62	0.75	7.26
	**NSm** ^**2**^	0.56	0.99	44.9	0.53	0.94	68.1	0.49	0.87	328.13	0.45	0.8	70.47	0.45	0.8	70.36
	**NGS**	0.77	0.91	3.5	0.63	0.74	5.16	0.62	0.73	312.33	0.58	0.68	5.39	0.61	0.72	5.23
	**PH**	0.67	0.96	3.3	0.39	0.56	10.16	0.4	0.57	73.1	0.38	0.54	10.16	0.4	0.57	10.14
	**GP**	0.68	0.88	0.6	0.47	0.61	2.05	0.46	0.59	24.3	0.43	0.56	2.06	0.43	0.56	2.05
	**Mean**	0.64	0.92	9.2	0.49	0.71	15.85	0.48	0.68	130.76	0.44	0.63	16.3	0.46	0.65	16.24
**Training WS and Testing WW**	**GY**	0.42	0.72	0.9	0.24	0.41	2.5	0.2	0.34	2.5	0.21	0.36	2.5	0.21	0.36	2.5
	**TKW**	0.85	0.99	2.5	0.65	0.76	7.59	0.6	0.7	7.67	0.6	0.7	7.7	0.62	0.72	7.64
	**NSm** ^**2**^	0.57	0.91	54.1	0.46	0.74	77.17	0.46	0.74	77.01	0.45	0.72	77.47	0.45	0.72	77.2
	**NGS**	0.7	0.81	4.3	0.66	0.77	5.44	0.64	0.74	5.56	0.59	0.69	5.83	0.63	0.73	5.65
	**PH**	0.72	0.87	2.9	0.52	0.63	9.97	0.42	0.51	10.06	0.4	0.48	10.07	0.45	0.54	10.04
	**GP**	0.7	0.76	0.6	0.53	0.57	2.04	0.47	0.51	2.05	0.43	0.47	2.06	0.46	0.5	2.06
	**Mean**	0.66	0.84	10.9	0.51	0.65	17.45	0.47	0.59	17.48	0.45	0.57	17.61	0.47	0.6	17.52
**Training CWR and Testing WS**	**GY**	0.59	1.32	0.6	0.65	1.46	1.3	0.61	1.36	0.61	0.6	1.34	1.33	0.6	1.34	1.33
	**TKW**	0.94	1.13	1.3	0.72	0.87	4.39	0.68	0.82	0.68	0.68	0.82	4.49	0.68	0.81	4.48
	**NSm** ^**2**^	0.74	1.31	37.9	0.71	1.25	49.98	0.7	1.24	0.7	0.67	1.18	52.72	0.67	1.18	52.24
	**NGS**	0.85	1.01	3.1	0.78	0.92	4.14	0.71	0.84	0.71	0.67	0.79	4.83	0.7	0.82	4.67
	**PH**	0.8	1.15	2.7	0.71	1.02	5.93	0.65	0.93	0.65	0.58	0.83	6.2	0.61	0.87	6.15
	**GP**	0.91	1.18	0.3	0.74	0.95	1.14	0.63	0.82	0.63	0.59	0.77	1.2	0.61	0.79	1.19
	**Mean**	0.81	1.18	7.65	0.72	1.08	11.15	0.66	1	0.66	0.63	0.96	11.8	0.64	0.97	11.68
**Training CWR and Testing WW**	**GY**	0.73	1.25	2.4	0.72	1.24	1.43	0.7	1.2	0.7	0.63	1.08	1.48	0.66	1.13	1.46
	**TKW**	0.96	1.11	8.5	0.77	0.89	4.81	0.68	0.79	0.68	0.65	0.76	5.16	0.67	0.78	5.1
	**NSm** ^**2**^	0.78	1.24	849.2	0.76	1.22	56.14	0.7	1.13	0.7	0.73	1.17	58.62	0.7	1.12	60.21
	**NGS**	0.86	0.99	16.1	0.73	0.85	4.91	0.75	0.87	0.75	0.73	0.85	4.97	0.76	0.88	4.8
	**PH**	0.84	1.01	11.4	0.77	0.93	5.7	0.71	0.86	0.71	0.66	0.79	6	0.67	0.81	5.95
	**GP**	0.92	1	5.5	0.78	0.84	1.14	0.71	0.77	0.71	0.66	0.71	1.19	0.67	0.73	1.18
	**Mean**	0.85	1.1	148.84	0.76	1	12.36	0.71	0.94	0.71	0.68	0.89	12.9	0.69	0.91	13.12

**Table:** 

	Traits	LASSO			RKHS			SVM			Extreme Gradient Boosting			RF		
		*r*_*MP*_	*r*_*MG*_	RMSE	*r*_*MP*_	*r*_*MG*_	RMSE	*r*_*MP*_	*r*_*MG*_	RMSE	*r*_*MP*_	*r*_*MG*_	RMSE	*r*_*MP*_	*r*_*MG*_	RMSE
**Training WW and Testing WS**	**GY**	0.2	0.45	2.41	0.24	0.54	2.41	0.32	0.72	2.5	0.35	0.78	2.5	0.37	0.83	2.4
	**TKW**	0.64	0.77	7.21	0.62	0.75	7.24	0.85	1.02	7	0.84	1.01	7	0.82	0.99	6.9
	**NSm** ^**2**^	0.5	0.88	68.86	0.47	0.83	69.72	0.58	1.03	64.1	0.56	0.99	75.2	0.58	1.03	66.9
	**NGS**	0.64	0.75	5.1	0.61	0.72	5.25	0.73	0.86	4.6	0.79	0.93	4.6	0.79	0.93	4.4
	**PH**	0.37	0.53	10.18	0.41	0.59	10.15	0.68	0.97	9.8	0.67	0.96	9.9	0.68	0.97	9.8
	**GP**	0.38	0.49	2.06	0.44	0.57	2.05	0.64	0.83	2.1	0.69	0.89	2	0.64	0.83	2
	**Mean**	0.46	0.65	15.97	0.47	0.67	16.14	0.63	0.91	15	0.65	0.93	16.9	0.65	0.93	15.4
**Training WS and Testing WW**	**GY**	0.19	0.33	2.5	0.21	0.36	2.5	0.38	0.65	2.5	0.35	0.6	2.5	0.4	0.69	2.5
	**TKW**	0.64	0.74	7.6	0.6	0.7	7.69	0.84	0.98	7.2	0.84	0.98	7	0.85	0.99	7.2
	**NSm** ^**2**^	0.5	0.8	75.51	0.46	0.74	77.25	0.59	0.94	73.6	0.56	0.9	75.2	0.6	0.96	72.5
	**NGS**	0.65	0.76	5.53	0.63	0.73	5.63	0.74	0.86	4.7	0.79	0.92	4.6	0.76	0.88	4.9
	**PH**	0.41	0.49	10.07	0.44	0.53	10.05	0.69	0.83	9.8	0.67	0.81	9.9	0.73	0.88	9.8
	**GP**	0.48	0.52	2.05	0.49	0.53	2.05	0.66	0.72	2	0.69	0.75	2	0.69	0.75	2
	**Mean**	0.48	0.61	17.21	0.47	0.6	17.53	0.65	0.83	16.6	0.65	0.83	16.9	0.67	0.86	16.5
**Training CWR and Testing WS**	**GY**	0.6	1.34	1.33	0.61	1.37	1.33	0.72	1.61	1.3	0.76	1.7	1.3	0.7	1.56	1.3
	**TKW**	0.69	0.84	4.47	0.7	0.84	4.44	0.91	1.1	3.7	0.95	1.14	3.5	0.91	1.09	3.8
	**NSm** ^**2**^	0.77	1.37	45.09	0.69	1.22	51.42	0.81	1.43	39	0.86	1.52	37.6	0.81	1.43	43.5
	**NGS**	0.75	0.89	4.35	0.71	0.84	4.56	0.88	1.03	3.2	0.94	1.11	2.3	0.91	1.07	3.4
	**PH**	0.61	0.88	6.16	0.65	0.93	6.09	0.87	1.24	5.2	0.92	1.31	5	0.85	1.21	5.3
	**GP**	0.62	0.81	1.19	0.63	0.82	1.18	0.85	1.1	1.1	0.92	1.19	1	0.86	1.12	1.1
	**Mean**	0.68	1.02	10.43	0.67	1	11.5	0.84	1.25	8.93	0.89	1.33	8.44	0.84	1.25	9.73
**Training CWR and Testing WW**	**GY**	0.61	1.05	1.49	0.67	1.15	1.46	0.8	1.38	1.3	0.87	1.5	1.3	0.83	1.42	1.4
	**TKW**	0.64	0.74	5.21	0.68	0.79	5.09	0.94	1.1	3.8	0.97	1.13	3.5	0.94	1.1	4.2
	**NSm** ^**2**^	0.77	1.24	55.17	0.71	1.13	60.01	0.85	1.36	47.8	0.9	1.45	37.6	0.85	1.36	49.3
	**NGS**	0.69	0.81	5.19	0.73	0.85	4.91	0.88	1.03	3.4	0.95	1.1	2.3	0.89	1.03	3.8
	**PH**	0.69	0.83	5.93	0.69	0.84	5.91	0.88	1.06	5.1	0.91	1.09	5	0.89	1.07	5.2
	**GP**	0.62	0.68	1.2	0.71	0.77	1.17	0.89	0.97	1	0.92	1	1	0.9	0.98	1.1
	**Mean**	0.67	0.89	12.36	0.7	0.92	13.09	0.87	1.15	10.41	0.92	1.21	8.44	0.88	1.16	10.82

WS (Water Stress), WW (Well-Watered), CWR (Combined Water Regimes - WS & WW); GY t/ha (Grain Yield), TKW (Thousand Kernel Weight), NSm2 (Number of Spikes per m²), NGS (Number of Grains per Spike), PH (Plant Height), GP (Grain Protein Content); RR-Blup -Ridge Regression Best Linear Unbiased Prediction; Bayes A, Bayes B, Bayes C, BRR (Bayesian Ridge Regression); LASSO (Least Absolute Shrinkage and Selection Operator); RKHS (Reproducing Kernel Hilbert Space Regression) RF-Random Forest; SVM- Support Vector Machine; XGB- Extreme Gradient Boosting; rMP - Predictive Ability; rMG -Prediction Accuracy; RMSE- Root Mean Square Error

## Discussion

Wheat production is challenged by environmental variations, particularly under rainfed systems resulting in lower and more variable yielding across years [56]. GS is a trending plant breeding method that can help enhancing yield and resilience more efficiently than traditional techniques. This study evaluates ten GS models including Bayesian, linear, ML, and kernel-based methods under WS, WW, and CWR conditions using two cross-validation strategies to identify the most accurate models for optimizing wheat breeding programs targeting Mediterranean environments and addressing production challenges.

### Impact of environmental conditions on heritability and genomic selection efficiency

Broad-sense heritability of key traits such as GY and TKW was higher under WW conditions compared to WS conditions. This increase can be attributed to higher variance of genotypes and reduced environmental variance under WW conditions, enabling genotypes to better express their genetic potential while reducing environmental influence on phenotypic expression. Consequently, heritability and GS accuracy improved in stable environments, enhancing the precision of selecting genotypes with desirable traits and increasing breeding efficiency [5, 57].

However, GS effectiveness depends on several factors, including the genetic architecture of the trait, training population size and composition, and marker density. GS generally achieves higher prediction accuracy for quantitative traits compared to marker-assisted selection (MAS), even with relatively low marker densities [5, 7]. Moreover, multivariate GS (MVGS) methods which account for correlations between traits, further enhance prediction accuracy [58].

In this study, GCV values were higher under WW conditions, suggesting greater genetic variability and selection potential in favourable environments. For example, GY genetic variation increased by 0.22 t/ha, and TKW by 3.51 g under irrigation (Table 2) highlighting the enhanced genetic potential available for selection.

Although PV exceeded GV for all traits, reflecting the significant influence of environmental factors, PV for GY decreased slightly (0.45 t/ha) under WW, suggesting greater environmental stability. Conversely, TKW PV increased marginally (8.20 g) under irrigation. These findings are consistent with Al-Naggar et al. [59], who reported higher selection gains under WW conditions. (Table 2). Overall, the higher heritability observed under WW conditions, driven by increased GV and reduced EV, underscores the importance of considering both variances in genomic selection strategies.

Under CWR, high genetic variability and heritability were also observed, particularly for grain weight and number traits. Notably, genotypic variance increased across all traits in CWR, for instance, GV for NSm² rose 724.6 nos compared with WS, while environmental variance decreased (e.g., EV for GY dropped from 0.24 t/ha in WS to 0.16 t/ha in CWR), and phenotypic variance remained relatively stable (e.g., PV for GY: 0.50 in WS vs. 0.48 in CWR). This balance between reduced environmental noise and enhanced genetic expression under CWR supports more consistent trait performance, reinforcing its suitability for genomic selection. This stability is particularly important for breeding programs aiming to improve yield consistency across different environments. By ensuring more accurate trait prediction this approach enhances breeding efficiency and ultimately accelerates genetic gains in wheat breeding programs.

### Yield-component trade-offs: insights for enhancing genomic selection models

The observed correlations provide valuable insights into the relationships among traits and their influence under WW conditions. The negative correlation between TKW and NGS under WW and WS conditions suggests a trade-off between grain size and grain number, which is a well-documented phenomenon in wheat and other cereals [60, 61]. As NGS increases, the assimilate distribution per grain decreases, leading to a reduction in TKW. Spike density (NSm²) demonstrated a strong positive correlation with GY under both WW and WS conditions, emphasizing its importance in yield formation. A higher spike density ensures greater grain production per unit area, which directly contributes to overall yield performance, underscores the importance of incorporating spike density in GS models. Thenegative correlation between GY and GP under both WW and WS conditions indicates a classic yield-protein trade-off, where an increase in GP often comes at the expense of yield due to competition for assimilates, emphasizing the need for GS model optimization to balance these competing traits. Cross-condition correlation analysis provides critical insights into genotype stability and the accuracy of GS models across varying water availability conditions. The moderate to strong correlations between WS and WW of GP (*r* = 0.69), NGS (*r* = 0.78), NSm² (*r* = 0.57), and PH (*r* = 0.67) suggest that these traits are relatively stable across environments, making them reliable targets for genomic selection. Notably, the strong correlation of TKW (*r* = 0.84) across conditions highlights its genetic consistency, reinforcing its potential as a key trait for selection. However, the low positive correlation of GY between conditions underscores its high environmental sensitivity, posing challenges for its prediction in GS models. The negative correlation between NGS under WS and TKW under WW suggests a trade-off between grain number and size across conditions, emphasizing the need for balanced selection strategies. Additionally, the negative correlation between GP (WS) with TKW and GY in WW highlights the complexity of maintaining both yield and quality in different environments.

### Influence of heritability, predictive ability, and cross-validation methods on genomic prediction accuracy across traits and environments

The relationship between heritability and *r*_*MP*_ can be complex. Lower heritability sometimes correlates with reduced *r*_*MP*_, particularly for traits like GY that are influenced by environmental variability [62]. However, some low-heritability traits can exhibit substantial or even higher *r*_*MP*_, especially with favorable genetic architecture or a well-structured training set [40, 63, 64]. This suggests that dividing *r*_*MP*_ by √h² can overestimate predictive ability, occasionally leading to biologically implausible *r*_*MG*_ values exceeding 1.0. Thus, both trait heritability and genetic architecture must be considered for reliable genomic prediction outcomes [65]. Although RRBLUP and BRR are both ridge-type models, our fits differ: RRBLUP was estimated by REML, whereas BRR used MCMC with conjugate priors (BGLR). In our high-dimensional settings ≫ (where *p* is number of genetic markers and *n* is genotyped/phenotyped individuals) inducing stronger shrinkage for BRR, explaining its slightly lower accuracies despite the methods’ conceptual similarity [66], Similar observations have been reported by Massman et al. [67] showing often negative values within testcross population in maize and Sirsat et al. [68] for most of their prediction models in wheat.

CV ensures generalizability by testing model on unseen data, but small sample sizes can lead to inconsistent predictions due to limited genetic representation [64, 69, 70]. Accordingly, larger and more representative samples are necessary for obtaining reliable performance estimates. KFCV and LOOCV provided stable estimates with lower variability, especially for RF and RR-BLUP. Meanwhile, LOOCV sometimes produced higher accuracy in selective conditions, particularly for GP, GY, TKW and increased variability in models sensitive to individual data points, such as XGB [71, 72].

This study revealed consistent results across most models and environmental conditions, with only slight differences between KFCV and LOOCV. For example, GP under WS conditions exhibited slight but consistent gains in prediction accuracy with LOOCV for both RR-BLUP (*r*_*MG*_ from 0.68 to 0.70) and RF (0.59 to 0.63), suggesting that finer, instance-level validation in LOOCV better captured genotypic effects for this trait. In addition, GY under WS showed a modest decline in RR-BLUP accuracy (from 0.75 to 0.69), possibly reflecting overfitting tendencies under KFCV due to heterogeneous training subsets in stress-prone environments. In contrast, XGB showed a marked improvement in TKW prediction under WW (*r*_*MG*_ from 0.41 to 0.69), with similar gains in WS and CWR, suggesting its strength in modeling complex trait-environment interactions under LOOCV. Likewise, PH prediction with RF improved in WW (*r*_*MG*_ from 0.63 to 0.76), indicating better generalization for stable physiological traits, suggesting trait-specific benefit of LOOCV [70]. Although RRBLUP consistently achieved high accuracy overall, other models demonstrated significant accuracy improvements with LOOCV for specific traits under both conditions (Fig. 2, Supplementary Table 1). Furthermore, with LOOCV, RF showed consistent performance across traits, reinforcing its reliability for genomic prediction and its potential for wheat breeding under varying water conditions. The variability in model performance across traits and validation methods emphasizes the importance of selecting appropriate validation strategies tailored to the trait and environmental context [73, 74]. This is especially relevant for traits influenced by complex G×E interactions, where LOOCV can provide more reliable and less biased evaluations, aiding model selection in genomic selection pipelines.

### Cross-condition genomic selection: evaluating model adaptability and transferability under contrasting water regimes

Cross-condition evaluation demonstrated the adaptability of GS models under varying environmental scenarios. Models trained on WW data underperformed when validated on WS conditions, reflecting the significant impact of environmental variability on prediction accuracy [16, 75]. This shortfall can be attributed to the lack of environmental stress representation in WW training data. Conversely, models trained on WS data and tested on WW data performed better, indicating adaptability to stable conditions [76, 77]. Although machine learning models were comparable to traditional approaches in predicting GY, they showed lower *r*_*MP*_ and higher RMSEs under WS, an outcome also observed in other traits. These results highlighted the challenge of transferring models from stable to variable environments. Nonetheless, ML models showed superior adaptability for GY compared to RR-BLUP, when trained on high-variability data (CWR), thereby enhancing the models’ ability to capture trait responses and improve predictive power in individual environment conditions. For example, when models trained on WW condition and testing on WS condition, RR-BLUP showed an increase in accuracy from 0.34 to 0.59, when trained on the mean data (an increase of 0.25). In contrast, XGB demonstrated a larger improvement, increasing from 0.35 to 0.76, yielding an improvement of 0.41. This stark difference highlights the exceptional ability of ML models like XGB to account for complex environmental interactions, making them valuable in challenging conditions (Table 5).

Traditional models like RR BLUP often struggle with high variability due to their linear assumptions and inability for modeling nonlinear relationships in complex traits [78]. While RR-BLUP performs well under controlled environments or smaller datasets, its effectiveness decreases when faced with greater variability, where the interplay of genetic factors becomes more pronounced [79]. In contrast, ML models, consistently managed these complexities, maintaining higher *r*_*MP*_ and lower error rates for traits like GY and NGSm^2^ indicative of their capacity to capture intricate genetic and environmental interactions [80, 81].

Previous research also confirms the variation in accuracies of ML models under diverse environmental conditions for rustresistance in wheat, alongside the effectiveness of deep learning approaches for handling environmental variability in durum wheat [34, 82]. Unlike traditional models with fixed parameters, ML models optimize performance by tuning hyperparameters, capturing complex nonlinear interactions between traits more effectively [83]. This flexibility contributes to their higher PAs and lower RMSEs, particularly when predicting across diverse environments.

One key finding is the improved accuracy of models trained on combined WW and WS data (CWR), as opposed to condition-specific datasets (WW or WS). Training on diverse datasets enhances generalization and robustness, allowing models to better capture the complexities of crop growth and yield dynamics across environments [80, 81]. For example, XGB consistently outperformed traditional models in predicting GY across both WW and WS conditions, emphasizing its practical reliability for crop breeding under climate variability [84].

Although Bayesian and kernel models showed moderate improvements when trained with mean datasets, they still fell short of RR BLUP (except when tested on WS) and ML models. This finding reinforces that while traditional approaches have merit, they may be insufficient for modeling the multifaceted interactions between environmental factors and crop traits. Differences in predictive accuracy between models trained on CWR and those trained under specific conditions emphasize the importance of robust training methodologies- such as updating the training population every cycle and including more environments to improve model transferability and predictive accuracy. The ability of ML models to account gene × gene and genotype × environment interactions position them as essential tools for future breeding research in the era of evolving agricultural practices [33, 85].

### Conclusions and implications for wheat breeding

Leveraging advanced GS approaches by integrating machine learning models with traditional methods enhances the capacity to capture complex interactions between traits and environmental factors. This strategy improves yield predictions under variable conditions, ensuring that models can more accurately reflect the challenges posed by both well-watered (WW) and water-stressed (WS) environments. Furthermore, using combined training datasets that encompass data from both WW and WS conditions significantly bolsters model robustness and generalizability, leading to more resilient and reliable breeding outcomes. In addition, optimizing trait selection by focusing on attributes with high stability-such as thousand kernel weight (TKW) and spike density-and carefully balancing trade-offs between yield and quality can drive more consistent genetic gains. By integrating these insights into breeding programs, the overall efficiency of the selection process is enhanced, accelerating the development of wheat varieties that are better adapted to Mediterranean and other challenging environments.

## Supplementary Information

Supplementary Material 1.

## Data Availability

The datasets generated during and/or analysed during the current study are available from the corresponding author on reasonable request.

## Abbreviations

CWR: Combined water regime
ECV: Environmental coefficient of variance
EV: Environmental variance
GCV: Genotypic coefficients of variation
GEBV: Genomic estimated breeding value
GEI: Genotype by environment interactions (GEI)
GP: Grain protein content
GS: Genomic selection
GV: Genotypic variance phenotypic variance (PV)
GY: Grain yield
KFCV: K-fold cross validation
LOOCV: Leave one out cross validation
MAS: Marker assisted selection
NGS: Number of grains perspike
NSm^2^: Number of spikes per unit area
PCV: Phenotypic coefficient of variation
PH: Plant height
RF: Random forest
RR-BLUP: Ridge regression best linear unbiased prediction
SVM: Support vector machine
TKW: Thousand kernel weight
TS: Training set
VS: Validation set
WS: Water stress
WW: Well-watered
XGB: Extreme gradient boosting
